# Poor Survivorship and Frequent Complications at a Median of 10 Years After Metal-on-Metal Hip Resurfacing Revision

**DOI:** 10.1007/s11999-016-4882-4

**Published:** 2016-05-17

**Authors:** Gulraj S. Matharu, Hemant G. Pandit, David W. Murray

**Affiliations:** Nuffield Department of Orthopaedics, Rheumatology and Musculoskeletal Sciences, Nuffield Orthopaedic Centre, University of Oxford, Oxford, OX3 7LD UK

## Abstract

**Background:**

High short-term failure rates have been reported for several metal-on-metal hip resurfacing (MoMHR) designs. Early observations suggested that MoMHRs revised to total hip arthroplasties (THAs) for pseudotumor had more major complications and inferior patient-reported outcomes compared with other revision indications. However, little is known about implant survivorship and patient-reported outcomes at more than 5 years after MoMHR revision.

**Questions/purposes:**

(1) What are the implant survivorship, proportion of complications and abnormal radiological findings, and patient-reported outcomes at a median of 10 years after MoMHR revision surgery? (2) Are survivorship, complications, and patient-reported outcomes influenced by revision indication? (3) Do any other factors predict survivorship, complications, and patient-reported outcomes?

**Methods:**

Between 1999 and 2008, 53 MoMHR revision procedures in 51 patients (mean age, 55 years; 62% female) were performed at one center and were all included in this retrospective study. Two patients (4%) were lost to followup and two patients (4%) died before a minimum followup of 7 years (median, 10.3 years; range 7–15 years). Revision indications included pseudotumor (n = 16), femoral neck fracture (n = 21), and other causes (n = 16). In most cases (62%, n = 33) both components were revised to a non-MoM bearing THA with the remainder (38%, n = 20: fracture, loosening, or head collapse) undergoing femoral-only revision to a large-diameter MoM THA. Postrevision complications, rerevision, Oxford Hip Score (OHS), and UCLA score were determined using both a longitudinally maintained institutional database and postal questionnaire. Implant survivorship was assessed using the Kaplan-Meier method (endpoint was rerevision surgery). Radiographs at latest followup were systematically assessed for any signs of failure (loosening, migration, osteolysis) by one observer blinded to all clinical information and not involved in the revision procedures.

**Results:**

Overall, 45% (24 of 53) experienced complications and 38% (20 of 53) underwent rerevision. Ten-year survival free from rerevision for revised MoMHRs was 63% (95% confidence interval [CI], 48%–74%). Revision indications were not associated with differences in the frequency of complications or repeat revisions. With the numbers available, 10-year survival free from rerevision for pseudotumor revisions (56%; 95% CI, 30%–76%) was not different from the fracture (68%; 95% CI, 42%–85%; p = 0.359) and other groups (63%; 95% CI, 35%–81%; p = 0.478). Pseudotumor revisions had inferior OHSs (median, 21; range, 2–46; p = 0.007) and UCLA scores (median, 2; range, 2–7; p = 0.0184) compared with fracture and other revisions. Ten-year survival free from rerevision after femoral-only revision using another large-diameter MoM bearing was lower (p = 0.0498) compared with all component revisions using non-MoM bearings. After controlling for potential confounding variables such as age, sex, and revision indication, we found femoral-only revision as the only factor predicting rerevision (hazard ratio, 5.7; 95% CI, 1.1–29; p = 0.040).

**Conclusions:**

Poor implant survivorship and frequent complications were observed at a median of 10 years after MoMHR revision. However, patients undergoing femoral-only revisions with large-diameter MoM bearings had the worst survivorship, whereas patients revised for pseudotumor had the most inferior patient-reported outcomes. Our findings suggest these two patient subgroups require regular surveillance after MoMHR revision.

**Level of Evidence:**

Level III, therapeutic study.

## Introduction

Frequent short-term failures have been observed with certain metal-on-metal hip resurfacing (MoMHR) designs with registries reporting 10-year revision rates between 10% and 13% [2, 30, 34]. Failure of MoMHRs may arise from traditional modes of arthroplasty failure (loosening, infection, dislocation), complications unique to hip resurfacing (femoral neck fracture and head collapse), and more recently from articulation-related problems [5, 6, 10, 17, 21, 31]. Previous reports have shown that MoMHRs revised for pseudotumor had an increased risk of major complications and inferior patient-reported outcomes at a mean 3-year followup compared with MoMHRs revised for other indications and matched primary THAs [13]. In addition to revision indication, other factors affecting implant survivorship and patient-reported outcomes after MoMHR revision are the type of revision performed and the articulation used [9, 10, 19, 26]. Furthermore, the type of revision performed and bearing used are interrelated factors affecting implant survivorship given femoral-only or acetabular-only revisions often result in large-diameter MoM THAs or MoMHRs, which are both associated with high failure rates [35, 36].

Many MoMHR designs were targeted for use specifically in young, active patients. Because many MoMHRs have been implanted worldwide and the high failure rates reported for several designs [16, 35], it is expected many patients will require revision surgery in the future. However, little is known about the frequency of complications, further surgery, and patient-reported outcomes more than 5 years postrevision [3, 14, 22, 26]. Furthermore, although factors such as revision indication, type of revision performed, and bearing surface have influenced short-term implant survivorship and patient-reported outcomes, it is unclear if these factors are important in predicting survivorship and patient-reported outcomes at extended followup.

We therefore sought to determine the following: (1) What are the implant survivorship, proportion of complications and abnormal radiological findings, and patient-reported outcomes at a median of 10 years after MoMHR revision surgery? (2) Are survivorship, complications, and patient-reported outcomes influenced by revision indication? (3) Do any other factors predict survivorship, complications, and patient-reported outcomes?

## Patients and Methods

This retrospective study was undertaken at one specialist arthroplasty center and is based on data from a longitudinally maintained institutional database. All patients were reviewed according to the institutional followup protocol; therefore, institutional review board approval was not required. All MoMHR revisions performed for any indication between December 1999 and March 2008 were eligible for inclusion regardless of where the primary MoMHR was performed.

Between December 1999 and March 2008, we performed 6664 primary THAs and 1249 revision THAs (most revisions were referred to our specialist unit). Of all primary THAs performed, 1308 (20%) were MoMHRs with the Birmingham Hip Resurfacing (BHR; Smith & Nephew, Warwick, UK; 48%, n = 633) representing the most commonly implanted MoMHR design. During this period, we used MoMHR in young active male and female patients with symptomatic hip arthritis. Of those who were treated with MoMHR, 24 patients (2%) died with their implant in situ, 167 (13%) MoMHRs were revised, and 37 (3%) were lost to followup, whereas 910 patients (1080 hips [82%]) were available for followup at a minimum of 2 years (median, 8.5 years; range, 2–16 years). We report on all MoMHR revisions performed for any indication between December 1999 and March 2008. These patients have previously been reported on at a mean of 3 years after MoMHR revision [13]. In addition to 49 MoMHR revisions of our own patients, we performed revisions on four patients referred from elsewhere. Of 51 patients (53 MoMHRs) undergoing revision, two patients (two hips [4%]) died with their revision implant in situ, 20 patients (20 hips [38%]) underwent rerevision, and two patients (two hips [4%]) were lost to followup, whereas 27 patients (29 hips [55%]) were available for followup at a minimum of 7 years (median, 10.3 years; range, 7–15 years). These cases represent the first 53 MoMHR revisions performed at this center and therefore include the surgeons’ learning curves with revising these implants.

Mean age at revision was 55 years (SD, 11 years) and 62% (n = 33) were women (Table 1). The most commonly revised implant was the BHR (55%, n = 29). Revisions were performed at a mean of 1.6 years (SD, 2 years) from the primary MoMHR by 13 surgeons. Revisions were performed for pseudotumor (30%, n = 16), femoral neck fracture (40%, n = 21), and other causes (30%, n = 16). Other causes included aseptic loosening (n = 8), femoral head collapse (n = 4), infection (n = 2), and recurrent dislocation (n = 2). Most revisions were performed through a posterior surgical approach (79%, n = 42; Table 1). In 62% (n = 33) of cases, both components were revised to a non-MoM bearing. All femoral stems were cemented (Exeter; Stryker, Newbury, UK; or CPT; Zimmer, Warsaw, IN, USA) and all acetabular components were uncemented (Trident [Stryker] or Trilogy [Zimmer]).

**Table 1:** Summary of 53 metal-on-metal hip resurfacings undergoing revision surgery

Patient and revision surgical factors	Details	53 hips (51 patients)
Sex	Male	20 (38%)
	Female	33 (62%)
Age at revision	Mean (range) in years	55.4 (23–71)
	SD	10.8
Body mass index at revision	Mean (range) in kg/m^2^	28.2 (19–39)
	SD	4.7
Bilateral metal-on-metal hips	Total patients	2 (4 hips)
Time to revision from hip resurfacing	Mean (range) in years	1.6 (0.01–7)
	SD	1.8
Resurfacing implant revised	Birmingham Hip Resurfacing (Smith & Nephew, Warwick, UK)	29 (55%)
	Conserve (Wright Medical Technology, Memphis, TN, USA)	21 (40%)
	Cormet (Corin, Cirencester, UK)	
		3 (6%)
Revision indication	Femoral neck fracture	21 (40%)
	Pseudotumor	16 (30%)
	Other	16 (30%)
Revision approach	Posterior	42 (79%)
	Anterolateral	8 (15%)
	Smith-Petersen	3 (6%)
Components revised	Both components	33 (62%)
	Femoral only^*^	20 (38%)
Stem fixation^†^	Cemented	48 (91%)
	Uncemented	5 (9%)
Revision femoral head diameter	Mean (range) in millimeters	38 (28–54)
	SD	8.5
Revision bearing	Metal-on-metal*	20 (38%)
	Metal-on-polyethylene	18 (34%)
	Ceramic-on-ceramic	10 (19%)
	Ceramic-on-polyethylene	5 (9%)

* All cases undergoing femoral-only component revision were revised to stemmed large-diameter metal-on-metal THAs; ^†^all acetabular fixation was uncemented.

In the remaining 38% (n = 20), only the femoral component was revised (typically for fracture, loosening, or head collapse) because the acetabular component was in an acceptable position intraoperatively and therefore retained. These 20 hips underwent femoral-only component revisions using either an uncemented (numerous different designs) or cemented stem (CPT) with large-diameter MoM bearings matching the inner diameter of the retained acetabular components. These revisions were performed pre-2008, before complications with large-diameter MoM THA bearings were known [35, 36].

All patients received antibiotic prophylaxis and thromboprophylaxis perioperatively and postoperatively according to the institution’s protocol at the time. Postoperative weightbearing was dependent on the reconstruction performed. Most patients were allowed to immediately bear full weight with walking aids as required. Up to 6 weeks of partial or nonweightbearing were recommended if concerns existed about the initial stability of the reconstruction and/or soft tissues after pseudotumor débridement. All patients received standard advice on antidislocation precautions for the first 6 weeks after revision (eg, sleep on back, avoid low chairs).

After revision, patients were reviewed in the clinic at 6 weeks and 1 year postoperatively. Thereafter review was according to clinical need, usually annually. Consultations included clinical examination, radiographs (AP pelvis and lateral hip), and completion of the Oxford Hip Score (OHS) questionnaire [7] and UCLA activity score questionnaire [1]. Patients with pain after revision underwent further investigation, including blood tests (to assess for infection and MoM bearing wear), cross-sectional imaging, and, where indicated, joint aspiration. From 2012 all patients with large-diameter MoM THAs underwent regular followup with blood metal ions and cross-sectional imaging as recommended by national authorities [28].

Study endpoints of interest after MoMHR revision were (1) complications related to surgery; (2) rerevision surgery; (3) OHS; (4) UCLA score; and (5) radiological abnormalities. One observer (GSM) searched the hospital’s clinical databases for all revised MoMHRs. Details of further surgery were recorded, including indication, surgical findings, and components exchanged. If further surgery was performed elsewhere, the respective hospital was contacted to complete data collection. Deaths were investigated using patient notes and information held by the general practitioner to determine whether deaths were related to surgery and whether rerevision occurred before death.

All surviving patients not undergoing rerevision received a postal questionnaire with nonresponders contacted by telephone (27 of 29 patients responded to the questionnaire [93%]). The questionnaire requested details of complications since revision, including further surgery. Patients also completed the OHS (0 = worst outcome and 48 = best outcome) [7, 29] and the UCLA activity score (1 = wholly inactive and 10 = regular participation in impact sports) [1]. All postrevision radiographs were assessed for evidence of component loosening, migration, or osteolysis [8, 11, 15] by one observer (GSM) blinded to all clinical information.

### Statistical Analysis

All statistical analysis was performed using Stata Version 13.1 (College Station, TX, USA). Either the median and range or the mean and SD were reported depending on data distribution. Survival analysis for revised MoMHRs was performed using the Kaplan-Meier method with rerevision surgery (removal or exchange of any component) used as the endpoint. Patients not undergoing further surgery were censored at latest followup (clinic review, questionnaire completion, or death).

The proportion of patients experiencing complications or undergoing rerevision was compared between different revision indications (pseudotumor, fracture, other) using a chi-square test with Yates’ correction. Differences in postrevision OHS and UCLA scores between revision indications were analyzed using the Kruskal-Wallis test. Cox proportional hazards models (univariate and multivariate) were used to assess the association of predictor variables (age, gender, body mass index, revision indication, type of revision performed, and bearing used) on time to rerevision. The proportional hazards assumption was assessed using Schoenfeld’s residuals. The significance level was set at p < 0.05 with confidence intervals (CIs) also at the 95% level.

## Results

**Fig. 1:**
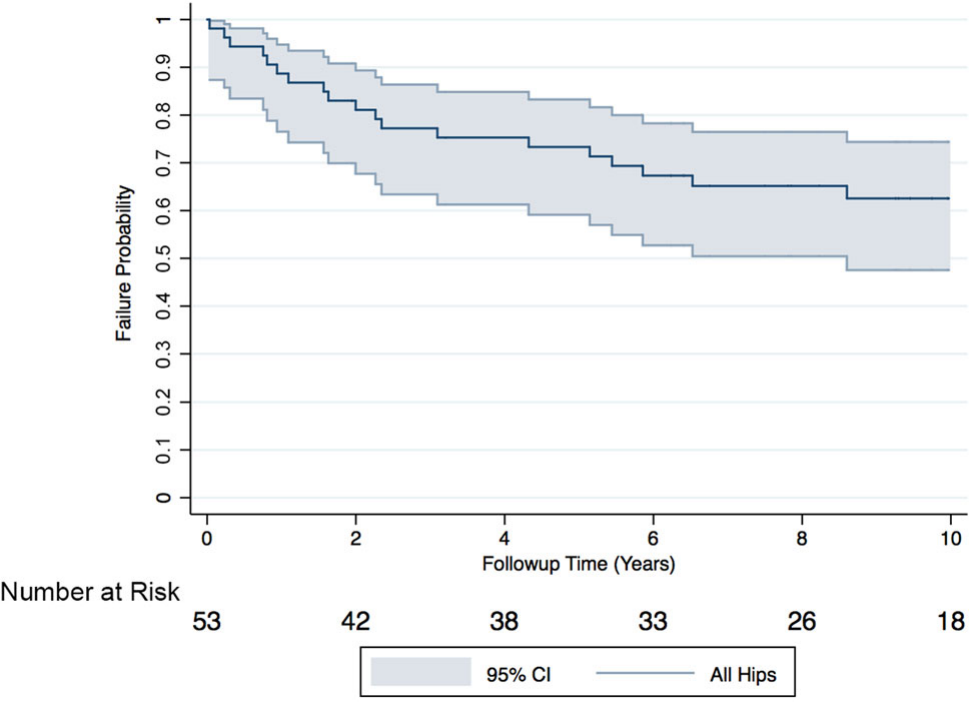
A Kaplan-Meier survival analysis illustrating the all-cause rerevision rate for 53 revised hip resurfacings at 10 years. The shaded area represents the upper and lower limits of the 95% CIs with the number of hips at risk detailed below the x-axis. The 10-year survival free from rerevision for revised hip resurfacings was 63% (95% CI, 48%–74%).

Overall, 45% (n = 24) of patients undergoing MoMHR revision surgery experienced complications and 38% (n = 20) underwent rerevision surgery. Rerevisions were performed at a mean 3 years (SD, 3 years) and were most commonly for pseudotumor (40%; n = 8), recurrent dislocation (20%; n = 4), and deep infection (20%; n = 4) (Table 2). Two patients (two hips) died at 6 years and 13 years after revision with no hip rerevised before death. Ten-year survival free from rerevision for all revised MoMHRs was 63% (95% CI, 48%–74%; Fig. 1). Median OHS and UCLA scores for surviving patients were 38 (range, 2–48) and 6 (range, 2–10), respectively. One surviving hip had evidence of radiological failure (stable nonprogressive radiolucency femoral Zone 1). Complications not resulting in rerevision included femoral nerve palsy (n = 3; two permanent and one resolved) and one case of symptomatic intermittent claudication caused by pseudotumor stenosis of the external iliac artery (required angioplasty and stenting).

**Table 2:** Clinical details of 20 revised metal-on-metal hip resurfacings requiring rerevision

Age at revision (years)/sex/HR design	Time to HR revision (years)	HR revision indication	Revision details	Time to rerevision (years)	Rerevision indication	Rerevision details	Outcome after rerevision
65/F/Conserve (Wright Medical Technology, Memphis, TN, USA)	0.1	Fracture	Stem-only revision (uncemented), MoM (44 mm)	0.03	Deep infection	Two-stage revision with uncemented cup, cemented stem, MoP	No complication at 10 years after rerevision
61/F/BHR (Smith & Nephew, Warwick, UK)	3.0	Pseudotumor	Uncemented cup, cemented stem, MoP (28 mm)	0.2	Recurrent dislocation	Exchange of head and liner, MoP	Two-stage rerevision for deep infection 2 years later
63/F/Conserve	1.2	Pseudotumor	Uncemented cup, cemented stem, CoC (32 mm)	0.3	Recurrent dislocation	Uncemented cup, cemented stem, constrained liner, MoP	Rerevision for recurrent dislocation (dual-mobility system + soft tissue reconstruction) + recurrent pseudotumor 5 years later
66/M/Conserve	0.9	AVN	Stem-only revision (cemented), MoM (54 mm)	0.8	Periprosthetic fracture	Stem-only revision (cemented), MoM, impaction grafting femur + wiring of fracture	Rerevision for massive pseudotumor (dual-mobility system + proximal femoral replacement) 7 years later
71/M/Conserve	0.1	Fracture	Stem-only revision (uncemented), MoM (50 mm)	0.8	Deep infection	Two-stage revision with uncemented cup, cemented stem, MoP	No complication at 9 years after rerevision
47/F/Conserve	1.9	Pseudotumor	Uncemented cup, cemented stem, MoP (32 mm)	0.9	Massive pseudotumor recurrence + dislocation	Pseudotumor débridement and exchange of head and liner, MoP	Multiple subsequent procedures 6 years later for pseudotumor recurrence, infection, and soft tissue repair
65/F/BHR	2.2	Pseudotumor	Uncemented cup, cemented stem, CoC (32 mm)	1.1	Recurrent dislocation	Exchange of head and liner, CoC	No further surgery; still has femoral nerve palsy and symptomatic intermittent claudication from external iliac artery stenosis
30/F/BHR	3.1	AVN	Stem-only revision (cemented), MoM (46 mm)	1.6	Pseudotumor	Pseudotumor débridement, uncemented cup, cemented stem, CoC	No complication at 7 years after rerevision
67/F/BHR	2.1	Pseudotumor	Uncemented cup, cemented stem, CoC (36 mm)	1.6	Aseptic loosening acetabular component	Femoral head allograft to acetabulum, cemented cup, CoC; femoral component retained	No complication at 7 years after rerevision
63/M/BHR	0.3	Fracture	Stem-only revision (uncemented), MoM (54 mm)	2.0	Deep infection	Two-stage revision with uncemented cup, uncemented stem, MoP	No complication at 10 years after rerevision
42/F/BHR	6.6	Pseudotumor	Uncemented cup, cemented stem, CoC (32 mm)	2.3	Aseptic loosening acetabular component	Uncemented cup, CoC; femoral component retained.	No complication at 5 years after rerevision
53/F/Cormet (Corin, Cirencester, UK)	6.6	Pseudotumor	Uncemented cup, cemented stem, MoP (28 mm)	2.4	Recurrent dislocation	Exchange of head and liner, MoP	Soft tissue reconstruction to improve hip stability 3 years later
23/F/BHR	3.0	Deep infection	Two-stage revision, uncemented cup, cemented stem, CoC (32 mm)	3.1	Deep infection	Two-stage revision, uncemented cup, cemented stem, CoC	Rerevision for dislocation 4 years later
49/F/BHR	3.5	Fracture	Stem only revision (cemented), MoM (42 mm)	4.3	Pseudo-tumour	Pseudotumor débridement, uncemented cup, cemented stem, CoC	No further surgery; stable femoral radiolucencies (Zones 2, 3, 7) at 1 year after rerevision
66/M/BHR	5.1	Pain + femoral neck impinging on cup	Stem-only revision (cemented), MoM (54 mm)	5.2	Pseudotumor	Pseudotumor débridement, uncemented cup, MoP; femoral component retained	No complication at 3 years after rerevision
57/M/BHR	5.2	Aseptic loosening femoral component	Stem-only revision (cemented), MoM (46 mm)	5.4	Pseudotumor	Pseudotumor débridement, femoral head allograft to acetabulum, uncemented cup, cemented stem, MoP	No complication at 2 years after rerevision
71/F/Conserve	0.1	Fracture	Stem-only revision (cemented), MoM (46 mm)	5.9	Pseudotumor	Pseudotumor débridement, uncemented cup, cemented stem, MoP	No complication at 1 year after rerevision
66/M/Conserve	3.1	AVN	Stem-only revision (cemented), MoM (46 mm)	6.5	Pseudotumor with dislocation	Pseudotumor débridement, uncemented cup, uncemented proximal femoral replacement, MoP	No complication at 1 year after rerevision
58/M/BHR	1.3	Fracture	Stem-only revision (cemented), MoM (50 mm)	8.6	Pseudotumor	Pseudotumor débridement, dual-mobility system, cemented stem, MoP; cup retained	No complication at 2 years after rerevision
48/F/BHR	0.2	Fracture	Uncemented cup, cemented stem, CoP (32 mm)	10.1	Periprosthetic fracture	Cemented femoral stem, MoP; cup retained	No complication at 2 years after rerevision

HR = hip resurfacing; F = female; M = male; BHR = Birmingham Hip Resurfacing; AVN = avascular necrosis; MoM = metal-on-metal; MoP = metal-on-polyethylene; CoC = ceramic-on-ceramic; CoP = ceramic-on-polyethylene.

**Fig. 2:**
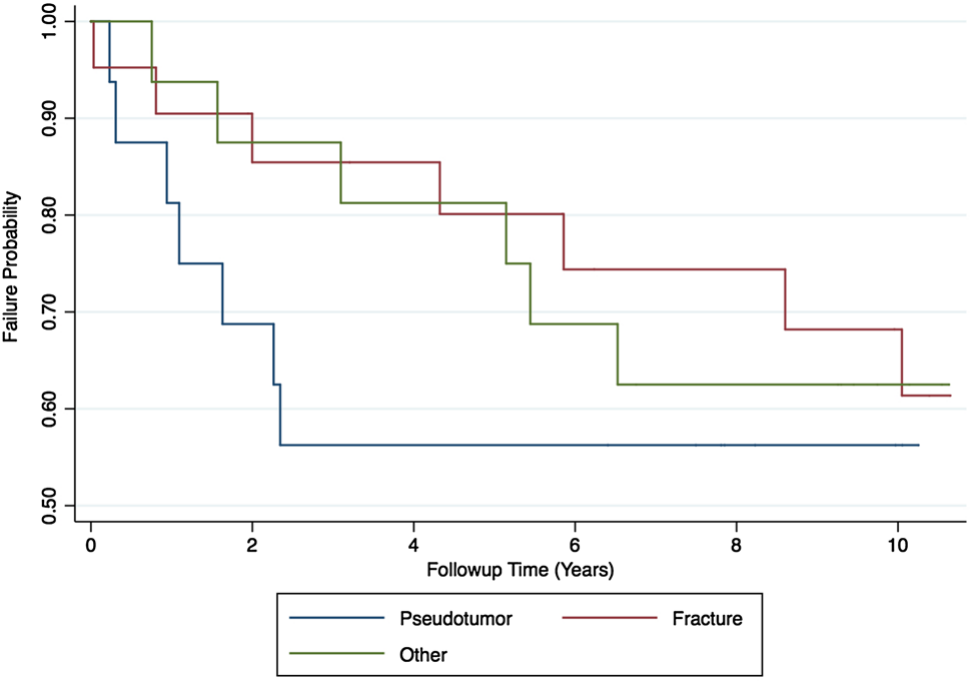
A Kaplan-Meier survival analysis illustrating the all-cause rerevision rate for 53 revised hip resurfacings at 10 years by initial revision indication. The CIs have not been included for clarity. Univariate analysis demonstrated 10-year survival free from rerevision after pseudotumor revision (56%; 95% CI, 30%–76%) was not different from the 10-year survival free from rerevision after revision for fracture (68%; 95% CI, 42%–85%; p = 0.359) and other indications (63%; 95% CI, 35%–81%; p = 0.478). This finding was confirmed in the multivariate model.

**Fig. 3:**
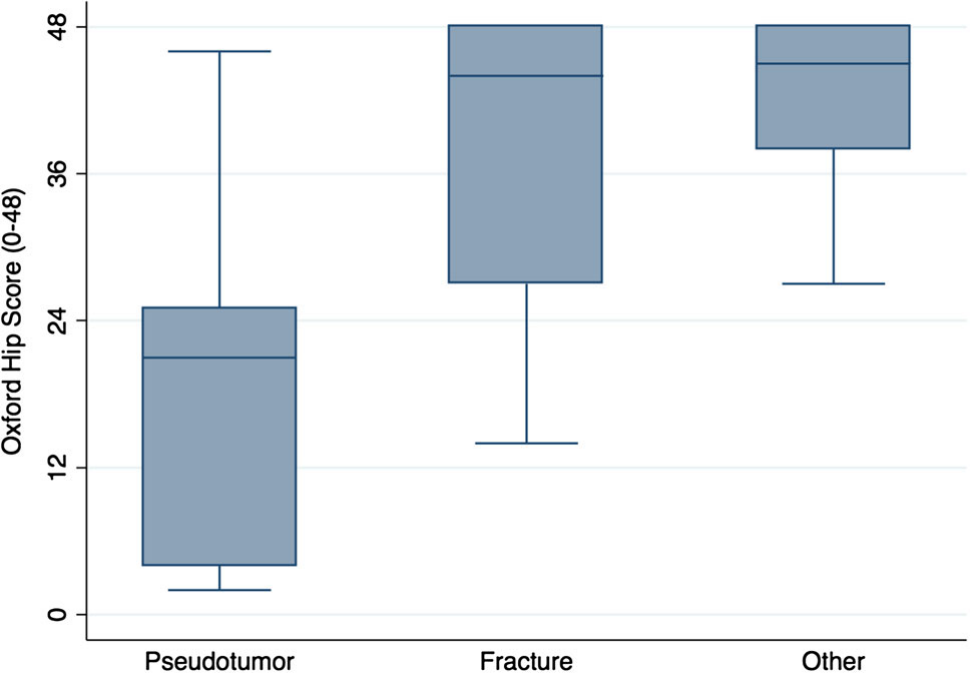
A box and whisker plot of the OHS after revision hip resurfacing by initial revision indication is shown. The horizontal line within the box is the median. The two ends of each box represent the 25th and 75th percentiles and the difference between these values is the interquartile range (IQR). The whiskers extending from the box represent the most extreme data points, which are no more than 1.5 times the IQR from the 75th percentile (upper whisker) and no less than 1.5 times the IQR from the 25th percentile (lower whisker). Values lying outside of the whiskers represent outliers.

**Fig. 4:**
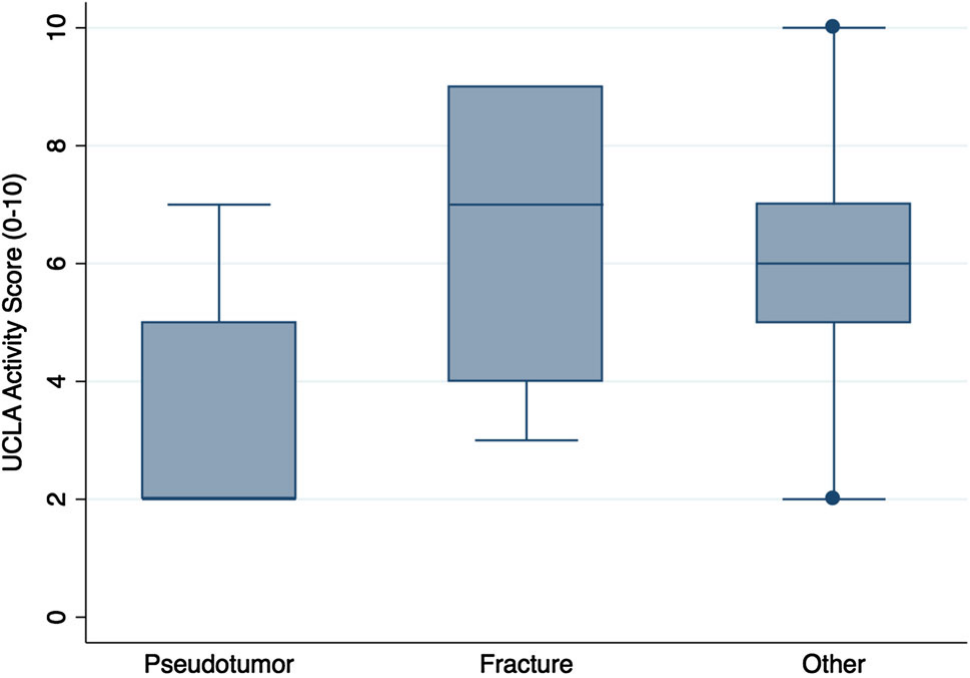
A box and whisker plot of the UCLA activity score after revision hip resurfacing by initial revision indication is shown.

The proportion of complications after pseudotumor revision (69% [11 of 16]) was no different with the numbers available compared with the fracture group (33% [seven of 21]) and the other group (38% [six of 16]) (chi-square test; p = 0.076; Table 3). The proportion of rerevisions after pseudotumor revision (44% [seven of 16]) was no different with the numbers available compared with the fracture group (33% [seven of 21]) and the other group (38% [six of 16]) (chi-square test; p = 0.811; Table 3). Ten-year survival free from rerevision after pseudotumor revision (56%; 95% CI, 30%–76%) was no different with the numbers available compared with that after revision for fracture (68%; 95% CI, 42%–85%; p = 0.359) and other indications (63%; 95% CI, 35%–81%; p = 0.478; Fig. 2). Patients undergoing pseudotumor revision had inferior OHSs (median, 21; range, 2–46; Kruskal-Wallis test; p = 0.007; Fig. 3) compared with patients revised for fracture (median, 44; range, 14–48) and other indications (median, 45; range, 27–48). Patients undergoing pseudotumor revisions had inferior UCLA scores (median, 2; range, 2–7; Kruskal-Wallis test; p = 0.0184; Fig. 4) compared with revisions for fracture (median, 7; range, 3–9) and other indications (median, 6; range, 2–10).

**Table 3:** Summary of complications and re-revisions by initial revision indication

Study outcome of interest after revision surgery	Whole cohort (n = 53)	Fracture group (n = 21)	Pseudotumor group (n = 16)	Other group (n = 16)
All complications	45% (n = 24)	33% (n = 7)	69% (n = 11)	38% (n = 6)
Complications not requiring rerevision surgery	8% (n = 4)	0% (n = 0)	25% (n = 4) 3 Femoral nerve palsy 1 Intermittent claudication resulting from stenosis of external iliac artery	0% (n = 0)
Complications requiring rerevision surgery	38% (n = 20)	33% (n = 7) 3 Pseudotumor 3 Deep infection 1 Periprosthetic fracture	44% (n = 7) 4 Recurrent dislocation 2 Acetabular component loosening 1 Pseudotumor recurrence with dislocation	38% (n = 6) 4 Pseudotumor 1 Deep infection 1 Periprosthetic fracture

**Fig. 5:**
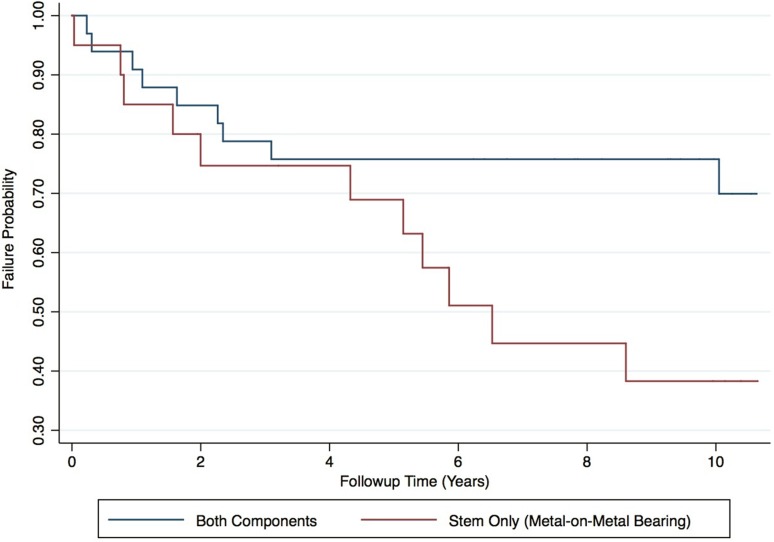
A Kaplan-Meier survival analysis illustrating the all-cause rerevision rate for 53 revised hip resurfacings at 10 years by type of revision performed. The CIs have not been included for clarity. Univariate analysis demonstrated 10-year survival free from rerevision after femoral-only revisions with another large-diameter MoM bearing (38%; 95% CI, 16%–60%) was lower (p = 0.0498) compared with all component revisions (76%; 95% CI, 57%–87%). This finding was confirmed in the multivariate model.

The proportion of rerevisions after femoral-only revision using another large-diameter MoM bearing (55% [11 of 20]) was higher compared with all component revisions using non-MoM bearings (27% [nine of 33]) (relative risk, 3.3; 95% CI, 1.2–8.9; p = 0.044). Ten-year survival free from rerevision after femoral-only revisions (38%; 95% CI, 16%–60%) was lower (p = 0.0498) compared with all component revisions (76%; 95% CI, 57%–87%; Fig. 5). After controlling for potential confounding variables such as age, sex, body mass index, and revision indication, we found that femoral-only revision was the only factor associated with an increased rerevision rate (hazard ratio, 5.7; 95% CI, 1.1–29; p = 0.040; Table 4). Of the 11 rerevisions performed after femoral-only revisions, 64% (n = 7) were for pseudotumor with the remainder performed for deep infection (27%, n = 3) and periprosthetic fracture (9%, n = 1). In hips not undergoing rerevision, there was no difference with the numbers available between the OHS (median, 41; range, 14–48 versus median, 37; range, 2–48; Wilcoxon rank-sum test p = 0.798) and UCLA score (median, 6; range, 3–9 versus median, 5.5; range, 2–10; Wilcoxon rank-sum test p = 0.425) in femoral-only revisions compared with all component revisions.

**Table 4:** Multivariate Cox proportional hazards model for identifying patients at risk of rerevision after revision of metal-on-metal hip resurfacings for all indications

Covariate	Multivariate analysis hazard ratio	p value
Sex, male versus female	0.87 (0.23–3.29)	0.837
Age at revision	1.02 (0.96–1.08)	0.497
Body mass index	1.05 (0.93–1.18)	0.461
Revision indication		
Pseudotumor versus	1.00	Baseline
Fracture	0.21 (0.31-1.40)	0.106
Other	0.31 (0.49–1.90)	0.203
Type of revision		
Femoral only with metal-on-metal bearing versus all component revision with nonmetal-on-metal bearing	5.65 (1.08–29.4)	**0.040**

Probability values < 0.05 (level of statistical significance) in bold; 95% confidence intervals for hazard ratios in parentheses.

## Discussion

Several MoMHR designs have experienced high short-term failure rates [35]. Studies reporting early implant survivorship and patient-reported outcomes after MoMHR revision observed inferior results after pseudotumor revisions [13, 26], single-component revisions [9, 10], and when using another MoM bearing [19, 26]. However, little is known about these endpoints more than 5 years after MoMHR revision. Because of high MoMHR failure rates and the young age of patients with these failed implants compared with patients undergoing THA revision [25, 32], it is important to establish long-term implant survivorship and patient-reported outcomes after revision so patients can be appropriately counseled about the results and potential risks of further intervention. This is the first study reporting on implant survivorship, the proportion of complications and abnormal radiological findings, and patient-reported outcomes at a median of 10 years after MoMHR revision. Although poor implant survivorship and frequent complications were observed after revision for all indications, pseudotumor revisions had inferior patient-reported outcomes compared with other revision indications. Furthermore, patients undergoing femoral-only revisions using large-diameter MoM bearings had the worst implant survivorship. These two patient subgroups therefore require regular followup after MoMHR revision.

We recognize limitations for our study. First, although our cohort size is similar to previous studies [9, 26, 37], some subgroup analyses may have been affected by small numbers. However, given the limited long-term data reported after MoMHR revision, we consider our findings important and recommend these can be used until larger studies become available. Second, we could not determine the latest implant status and patient-reported outcomes in two hips (censored between 2 and 6 years postrevision). These patients may have undergone rerevision elsewhere or may have unsatisfactory patient-reported outcomes, which would affect our findings. Third, this study includes the surgeons’ learning curves with revising MoMHRs. This may have adversely affected the reported findings, because increasing experience improves implant survivorship and patient-reported outcomes after MoMHR revision [9]. Finally, our findings may not apply after revision of MoM THAs or MoMHR designs not assessed here.

Almost half of the patients undergoing MoMHR revision subsequently experienced major complications with over one-third undergoing rerevision. Early studies reported short-term survivorship and patient-reported outcomes after MoMHR revision for most indications were comparable to conventional THA [3, 13, 22]. Our findings suggest this is not true at long-term followup. Although our surviving MoMHR revisions have OHSs (median, 38) similar to those reported 10 years after primary THA (median, 41) [23], the 10-year survival free from rerevision for revised MoMHRs (63%) was considerably lower compared with primary THA (96%–98%) [30]. Perhaps more concerning is that 10-year survival free from rerevision for revised MoMHRs is lower than that for the recalled Articular Surface Replacement (DePuy, Warsaw, IN, USA) MoMHR device (72%; 95% CI, 70%–74%) [27, 28, 30]. This illustrates how poor the long-term results of MoMHR revision can be. It is important for surgeons to appreciate this poor implant survivorship and for young MoMHR patients potentially undergoing further surgery to be made aware of this. Importantly, all operations were performed by experienced revision surgeons previously reporting 10-year survival free from rerevision of 82% (95% CI, 80%–85%) after 1176 revision THAs [32]. Therefore, although there may be a learning curve for revising MoMHRs, our findings suggest these revisions must not be underestimated and considered “simple” revisions, even for experienced arthroplasty surgeons. These poor results raise the question of whether surgeons should consider early revision with some evidence suggesting this improves short-term survivorship and patient-reported outcomes [9].

An earlier report on this cohort observed pseudotumor revisions had a higher proportion of complications and inferior patient-reported outcomes compared with MoMHRs revised for other indications [13]. More recently Su and Su [37] reported inferior patient-reported outcomes in MoMHRs revised for unexplained pain compared with other indications. The present study observed no difference in the proportion of complications or implant survivorship in MoMHRs revised for pseudotumor compared with other indications. We suspect this variance with our earlier report [13] relates to followup length. All pseudotumor revisions undergoing rerevision occurred within 3 years of initial revision (Fig. 2) when the survival free from rerevision of nonpseudotumor revisions was acceptable. The poor initial survivorship and patient-reported outcomes after pseudotumor revision are likely related to intervening at a late stage because at the time the destructive nature of pseudotumors was not appreciated [13, 31]. Between 3 and 10 years after initial revision, a number of the fracture and other MoMHR revisions underwent rerevision (Table 2). Most rerevisions in the fracture and other groups were also for pseudotumor because these patients were initially revised to large-diameter MoM THAs at a time when the high failure rates of such devices were unknown [36]. Therefore, we suspect that MoMHR revisions for nonpseudotumor indications (fracture, loosening, head collapse) would have achieved better survivorship and/or patient-reported outcomes than those reported if they were not initially revised to another large-diameter MoM bearing. By contrast, our observations that patient-reported outcomes were inferior in surviving pseudotumor revisions compared with MoMHR revisions for other indications support short-term reports [13, 26, 37]. Although the patient-reported outcomes at a median of 10 years after MoMHR revision for fracture and other indications are comparable to primary THA [23], patient-reported outcomes after pseudotumor revision remained poor. The poor patient-reported outcome after pseudotumor revision is again related to our late recognition of these first 16 pseudotumors, which can represent a destructive complication [13, 19, 31]. In all 16 pseudotumor revisions, there was some degree of macroscopic damage to the gluteus medius muscle with or without associated bone loss with three cases also having more extensive damage of the surrounding soft tissues, which required reconstructive input from a plastic surgeon at revision. Furthermore, débridement of affected periprosthetic tissues can adversely affect hip stability and patient-reported outcomes [24]. Although our pseudotumor revisions were performed when this complication was not understood, increased awareness has resulted in improved patient-reported outcomes after pseudotumor revision at this center [20] and elsewhere [9].

Femoral-only revision with large-diameter MoM bearings was the only predictor of rerevision. Even when revision indication was controlled for, these patients had an increased rerevision risk (hazard ratio, 5.7) compared with all component revisions using non-MoM articulations. The poor implant survivorship after femoral-only revisions was mainly the result of pseudotumor formation. All femoral-only revisions were performed in MoMHRs initially revised for fracture or other reasons; therefore, no pseudotumors were present at the first revision procedure. Most rerevisions in this subgroup were performed for new pseudotumors, which developed after implantation of the second large-diameter MoM bearing. Our findings support earlier observations that single-component MoMHR revisions to another MoM bearing result in inferior survivorship and patient-reported outcomes compared with all component revisions using non-MoM articulations [9, 10, 19]. These poor results are related to the use of large-diameter MoM THAs as the revision implant with high failure rates reported for these devices [4, 16, 36], resulting from pseudotumor formation because of wear and corrosion at the stem taper and femoral head interface as well as at the bearing surface [18]. Our revisions were performed before recognition of this problem. Although most regulatory authorities recommend regular followup of large-diameter MoM THAs regardless of symptoms [12, 28, 38], it is important not to overlook patients who currently have these implants in situ after early single-component MoMHR revisions. Our observations suggest this subgroup requires regular surveillance as a result of the increased risk of developing pseudotumors within 10 years of revision. In light of our findings, it is now our preference to revise both MoMHR components and implant a non-MoM THA when dealing with failed MoMHRs. This has been suggested previously [19, 26], and using this approach in a subgroup of our patients produced a more acceptable 10-year survival rate free from rerevision of 76% after revision MoMHR. However, we acknowledge that other surgical options exist. Recently some surgeons have elected to retain well-fixed and adequately positioned acetabular components when revising failed MoMHRs. These procedures involve single-component revisions using dual-mobility metal-on-polyethylene articulations or a polyethylene liner cemented into the retained MoMHR shell with good subsequent implant survivorship and patient-reported outcomes observed [33]. Although this may become a more acceptable approach for failed MoMHRs in the future, it must be recognized that only short-term results have been reported and that the use of such techniques is currently off-label [33].

Poor implant survivorship and frequent complications were observed at a median of 10 years after MoMHR revision surgery performed for all indications. However, patients undergoing femoral-only revisions with large-diameter MoM THA bearings had the worst survivorship with these patients almost six times more likely to undergo rerevision compared with all component revisions using non-MoM bearings. Furthermore, patients revised for pseudotumor had inferior patient-reported outcomes compared with MoMHRs revised for other indications. We therefore recommend these two patient subgroups undergo regular clinical surveillance (typically annually) in line with previously published followup protocols [12, 38].
